# Supplementary material for: Identification of NOX4 as a New Biomarker in Hepatocellular Carcinoma and Its Effect on Sorafenib Therapy
Source: Biomedicines. 2023 Aug 4;11(8):2196. doi: 10.3390/biomedicines11082196 (PMC10452076; doi:10.3390/biomedicines11082196)
Supplement: Supplementary file 1 [file biomedicines-11-02196-s001.zip › biomedicines-2497642-supplementary.pdf]

Supplementary Materials

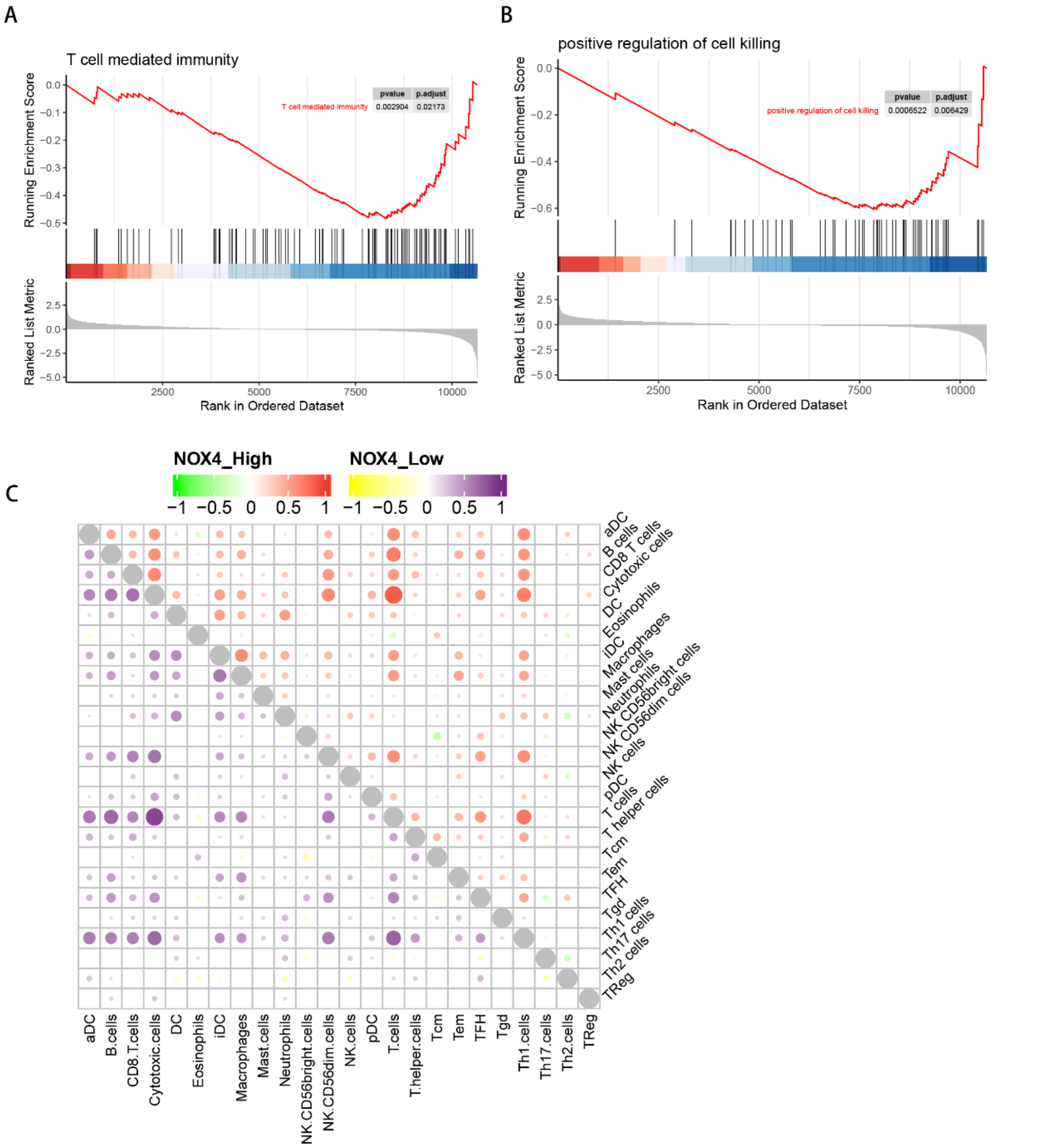

Figure S1 (A-B) The GSEA enrichment showed two significantly signaling pathways including T cell mediated immunity (A) and positive regulation of cell killing (B). (C) Microenvironmental immune cell profiling of NOX4-High expression and NOX4-Low expression HCC.

A

	NOX4-HIGH (n=86)	NOX4-LOW (n=64)
Median OS (months)	30	102
Median PFS (months)	9	45

B

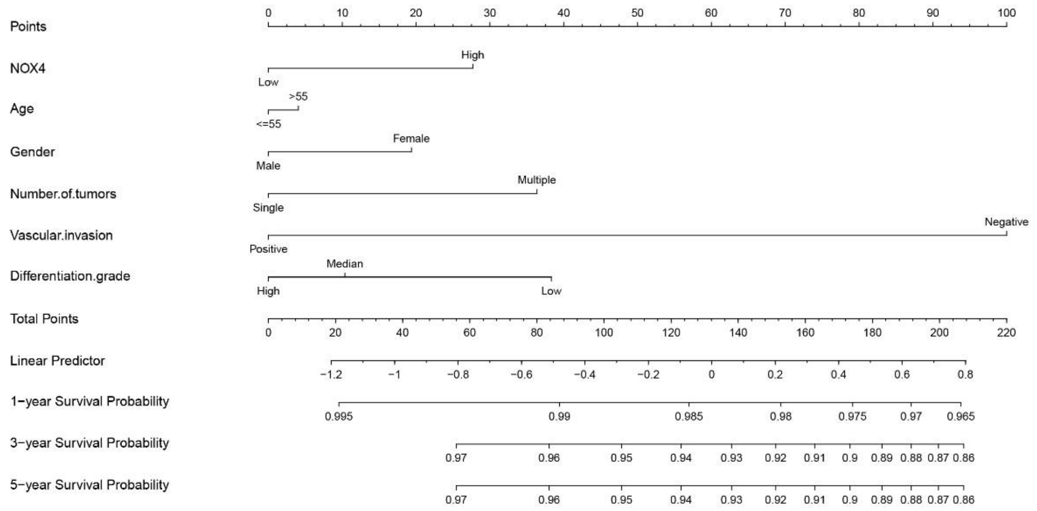

Figure S2 (A) The median survival time of high (n = 86) and low (n = 64) NOX4 expression groups. (B) ROC of the NOX4 nomogram for predicting the probability of 1-, 3- and 5- years OS in the Xiangya cohort. Nomogram constructed combined with NOX4 and other risk factors (AGTVG).

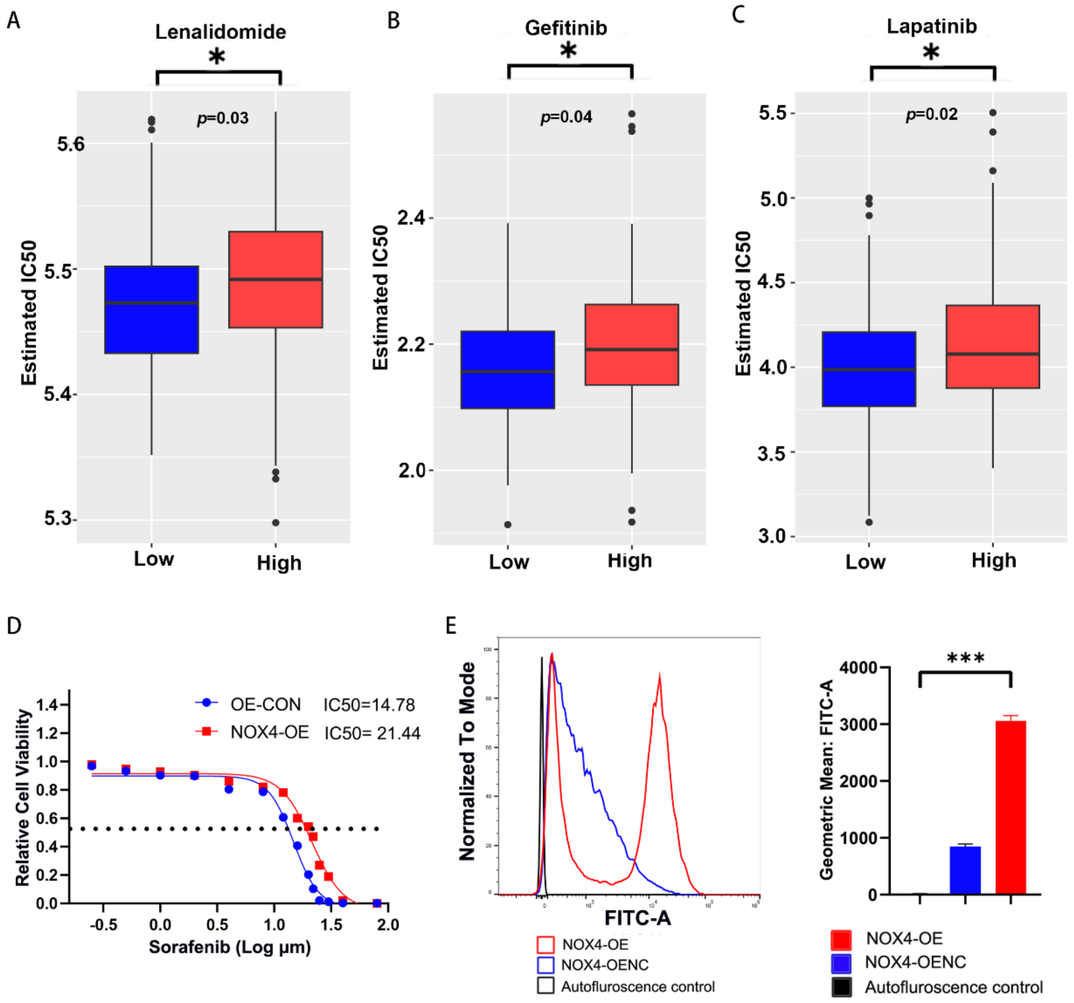

Figure S3 Differential putative chemotherapeutic and immunotherapeutic response. (A-C) The box plots of the estimated IC50 for Lenalidomide(A), Gefitinib (B) and Lapatinib (C) is shown in NOX4-high and NOX4-low expression in LIHC in TCGA cohort. (D) The IC50 of NOX4-OE and OE-CON in HepG2 cells after treated with sorafenib for 72 h. (E) Representative histograms for the fluorescence induced by DCFH-DA probe in NOX4-OE and OE-CON in HepG2 cells. (*P < 0.05, **P < 0.01, and ***P < 0.001).
